# Timing over Dose: Maternal Vitamin D, Periconceptional Window, and Early-Life Respiratory Programming

**DOI:** 10.3390/nu18142333

**Published:** 2026-07-16

**Authors:** Oana Raluca Temneanu, Adriana Mihai, Andreea-Luciana Avasiloaiei, Alina Murgu, Vasile Valeriu Lupu, Ancuța Lupu, Felicia Trofin, Ileana Ioniuc, Emil Anton, Luiza-Simona Pohaci-Antonesei, Otilia Novac, Manuela Ștefan, Bianca Simionescu

**Affiliations:** 1 Grigore T. Popa University of Medicine and Pharmacy, 700115 Iași, Romania; temneanu.oana@umfiasi.ro (O.R.T.); andreea.avasiloaiei@umfiasi.ro (A.-L.A.); alina.murgu@umfiasi.ro (A.M.); vasile.lupu@umfiasi.ro (V.V.L.); ancuta.ignat1@umfiasi.ro (A.L.); felicia.trofin@umfiasi.ro (F.T.); ileana.ioniuc@umfiasi.ro (I.I.); emil.anton@umfiasi.ro (E.A.); pohaci.antonesei@umfiasi.ro (L.-S.P.-A.); otilia.novac@umfiasi.ro (O.N.); stefan.manuela@umfiasi.ro (M.Ș.); 2 Iuliu Hațieganu University of Medicine and Pharmacy, 400012 Cluj-Napoca, Romania; bianca.simionescu@umfcluj.ro

**Keywords:** maternal nutrition, vitamin D, 25-hydroxyvitamin D, placental biology, decidua, early-life programming, periconceptional window, foetal lung development, childhood asthma, 17q21, prenatal exposome, precision prevention

## Abstract

**Background:** Vitamin D deficiency affects an estimated 40–60% of pregnant women worldwide and is associated with adverse obstetric and neonatal outcomes. Childhood asthma, the most prevalent chronic paediatric disease, has emerged as a plausible programming target, since vitamin D regulates foetal lung branching morphogenesis, calibrates the developing immune system, and modulates decidual and placental function in early gestation. Two landmark randomised trials, VDAART (intervention from weeks 10–18) and COPSAC2010 (from week 24), each reported a 20–25% reduction in offspring asthma or recurrent wheeze at age 3, yet neither reached significance in primary analysis, and the protective signal attenuated by school age. Post hoc stratification by baseline maternal 25-hydroxyvitamin D [25(OH)D] and 17q21 genotype recovered significant effects, raising the possibility that population-average nulls conceal a real but modifier-conditional benefit. **Aim:** This narrative review re-examines the evidence through a developmental-timing lens, arguing that the periconceptional and first-trimester window, rather than mid-gestation, is the biologically relevant interval for any protective effect. **Methods:** The review utilises a narrative synthesis of randomised trials, birth-cohort studies, mechanistic investigations, and recent meta-analyses (PubMed, Embase, Cochrane Library to April 2026) relevant to maternal vitamin D, placental biology, and offspring asthma. **Findings:** The periconceptional weeks coincide with implantation, decidualisation, the embryonic and pseudoglandular phases of airway morphogenesis, and the onset of epigenetic programming, while decidual CYP27B1 expression is prominent in the first trimester. Both trials initiated supplementation after branching morphogenesis was largely complete. Effect modifiers, including baseline 25(OH)D, vitamin D-binding protein, and maternal 17q21 genotype, indicate substantial inter-individual heterogeneity masked in unselected populations. **Conclusions:** Repositioning preventive supplementation toward the preconceptional and first-trimester window, stratified by baseline status, offers a biologically coherent strategy that existing mid-pregnancy trials have not tested. Adequately powered preconceptional trials with serial biomarker measurement and objective respiratory phenotyping are the priority.

## 1. Introduction

Asthma is the most common chronic disease of childhood, and its origins are increasingly traced to prenatal and early-life exposures rather than to postnatal triggers alone. Established prenatal and perinatal risk factors include maternal smoking and gestational exposure to ambient air pollution, maternal atopy and asthma, caesarean delivery, early-life respiratory viral infection, and disrupted microbial exposure, alongside nutritional influences [1,2]. The developmental origins of health and disease (DOHaD) framework, articulated by Barker and extended by Gluckman and colleagues, established that nutritional and environmental conditions during the first 1000 days, from conception to the second birthday, exert effects on adult disease risk that later interventions cannot replicate [3,4]. Suboptimal nutrition in this window has been estimated to contribute to a substantial fraction of the global burden of childhood mortality and non-communicable disease [5]. Among the candidate prenatal determinants of respiratory health, maternal vitamin D status has attracted sustained interest: vitamin D regulates foetal lung branching morphogenesis, modulates the developing immune system, and shapes the maternal–foetal interface, providing a plausible biological route from maternal nutritional status to offspring airway disease. However, whether optimising maternal vitamin D can prevent childhood asthma has proven to be one of the more frustrating questions in perinatal nutrition: it is rich in mechanistic promise but equivocal in trial confirmation.

The contemporary evidence rests on two large randomised trials, both reported in 2016. Litonjua et al. found, in the Vitamin D Antenatal Asthma Reduction Trial (VDAART), that 4400 IU/day cholecalciferol from weeks 10–18 reduced offspring asthma or recurrent wheeze at age 3 (hazard ratio 0.8, 95% CI 0.6–1.0, *p* = 0.051) [3]. Chawes et al. found, in the Copenhagen Prospective Studies on Asthma in Childhood 2010 (COPSAC2010), that 2800 IU/day cholecalciferol from week 24 produced persistent wheeze in 16% of children versus 20% on standard dose (hazard ratio 0.76, 95% CI 0.52–1.12, *p* = 0.16) [4]. Their patient-level combined analysis estimated an adjusted odds ratio for asthma or recurrent wheeze at 0–3 years of 0.74 (95% CI 0.57–0.96, *p* = 0.02), with the effect concentrated in women whose entry 25(OH)D was already ≥30 ng/mL (≥75 nmol/L) (aOR 0.54, 95% CI 0.33–0.88, *p* = 0.01) [5].

However, the protective effect proved transient and population-dependent rather than simply fragile. The VDAART 6-year follow-up found no preserved difference between treatment arms [6]; COPSAC2010 reproduced the same attenuation [7]. The 15-year synthesis from the VDAART group recovered protection at ages 3 and 6 once analyses were adjusted for entry 25(OH)D and stratified by maternal genotype at 17q21 rs12936231 [8]. The 2025 Cochrane update by Patchen et al. concluded that high-dose vitamin D in pregnancy likely reduces childhood wheeze (RR 0.79, 95% CI 0.64–0.98; three studies, 1439 participants; moderate-certainty evidence) but produces little to no difference in childhood asthma per se (RR 0.81, 95% CI 0.63–1.04) [6]. The 2025 meta-analyses extended this picture. Li et al., pooling seven randomised trials with 3958 mother–infant pairs, found no significant overall effect on respiratory or allergic outcomes [7]. Shen et al. reached convergent conclusions across an overlapping but non-identical evidence base, reinforcing the view that mid-pregnancy supplementation confers, at most, a small and non-durable reduction in childhood wheeze [8].

Two readings of this evidence are possible. The first, taken at face value, invites scepticism: maternal vitamin D supplementation in mid-pregnancy may not prevent school-age asthma in unselected populations. The second asks a more demanding question: if the biology linking vitamin D to airway programming is real, and the mechanistic, observational, and post hoc trial data suggest that it is, why does an intervention beginning at weeks 10–18 yield only a transient effect? The answer defended below is one of timing rather than dose, since the most vitamin D-sensitive phases of airway morphogenesis precede the trials’ start.

Embryonic lung development begins in the fourth post-conceptional week, with bifurcation of the laryngotracheal groove and the appearance of primary bronchial buds; the conducting airway tree is then established by branching morphogenesis, predominantly during the pseudoglandular stage that extends from approximately week 5 to week 17 [9]. Endometrial vitamin D receptor (VDR) expression and decidual 1α-hydroxylase activity precede implantation, and decidual CYP27B1 is demonstrably active from the earliest weeks of gestation [10,11]. Therefore, maternal 25(OH)D status during the periconceptional weeks conditions the molecular environment in which the embryonic airway is patterned, an interval that observational cohorts rarely sample and that no completed randomised trial has targeted.

This review synthesises the evidence on maternal vitamin D from the months preceding conception through the end of the first trimester, placing the placenta and decidual interface at the centre of the mechanistic account. It develops three propositions, stated here explicitly. First, the failure of mid-gestational supplementation to produce durable protection is compatible with an intervention delivered after the critical developmental window has closed and after the placental–decidual programme is largely set. Second, the modifiers identified in post hoc analyses (baseline 25(OH)D, vitamin D-binding protein (DBP), genotypes at the GC (encoding DBP), VDR, and 17q21 loci, and interactions with the broader prenatal exposome) indicate that a one-size-fits-all strategy is unlikely to succeed. Third, the precedent of periconceptional folate supplementation for neural tube defect prevention offers a translational model for what a preconception vitamin D strategy could resemble. Definitive randomised evidence for preconceptional intervention does not yet exist, so the argument is exploratory. The synthesis is narrative in scope, drawing selectively on the randomised trials, birth cohorts, mechanistic studies, and recent meta-analyses most relevant to the periconceptional question rather than on a systematic protocol-driven search.

### Literature Search, Evidence Selection, and Terminology

Because the questions pursued here are mechanistic and hypothesis-generating rather than quantitative, the review is narrative, and the search that supports it was designed to map the relevant evidence rather than to underpin a pooled estimate. PubMed, Embase, and the Cochrane Library were searched from database inception to April 2026. Search strings combined controlled vocabulary and free-text terms for the exposure (“vitamin D”, “25-hydroxyvitamin D”, “cholecalciferol”, “vitamin D-binding protein”), the reproductive interval (“pregnancy”, “periconception”, “preconception”, “first trimester”, “placenta”, “decidua”) and the outcomes of interest (“asthma”, “wheeze”, “respiratory”, “lung development”, “immune programming”), joined by the Boolean operators AND, OR. Reference lists of the retrieved trials, cohorts, and reviews were screened by hand, and a small number of foundational mechanistic papers known to the authors were added where the database search had not surfaced them.

Eligible sources were randomised controlled trials, prospective and retrospective birth-cohort studies, mechanistic investigations in human and animal tissue, and systematic reviews or meta-analyses, restricted to English-language publications. Case reports, conference abstracts without full data, and studies without a respiratory, immunological, or placental outcome were not considered. Where the evidence conflicted—and on several questions it did—priority was given to randomised trials and to recent meta-analyses over observational associations, and to studies that measured 25(OH)D by liquid chromatography–tandem mass spectrometry over those relying on immunoassay. Post hoc and subgroup analyses were retained where they were biologically informative but flagged throughout as hypothesis-generating rather than confirmatory, since their interpretation is constrained by the multiplicity of comparisons and by residual confounding. No formal risk-of-bias scoring or quantitative synthesis was undertaken, consistent with the narrative aim; the trade-off is transparency of reasoning at the cost of the reproducibility a systematic protocol would confer.

Four temporal terms recur in what follows, and because the argument turns on timing, they are worth fixing at the outset. “Preconceptional” denotes the interval before fertilisation, typically the weeks to months during which maternal vitamin D status is established before a pregnancy begins. “Periconceptional” is used more broadly, spanning the weeks on either side of conception, from roughly four weeks before to ten weeks after the last menstrual period, and therefore encompassing implantation, decidualisation, and the earliest phases of organogenesis. “First trimester” refers to the conventional obstetric window from conception to the end of gestational week 13. “Early gestational” is the least precise of the four and is reserved for statements that apply across the first trimester and the beginning of the second without implying a sharper boundary. The periconceptional and first-trimester windows overlap substantially, and it is their conjunction, rather than any single week, that the developmental-timing hypothesis identifies as decisive.

## 2. Vitamin D, the Placenta, and the Decidual Interface in Early Pregnancy

Vitamin D is a steroid prohormone rather than a vitamin in the classical sense. The active metabolite 1,25-dihydroxyvitamin D [1,25(OH)_2_D] binds the vitamin D receptor (VDR), a nuclear transcription factor expressed across many tissues, including endometrial stroma, decidua, syncytiotrophoblast, ovary, and testis [11,12]. CYP27B1, the 1α-hydroxylase that converts 25(OH)D to active hormone, is expressed in deciduo-trophoblastic tissue and produces 1,25(OH)_2_D locally during early pregnancy [10]. Therefore, the placenta functions not only as a passive conduit for maternal 25(OH)D but as an active endocrine organ that generates 1,25(OH)_2_D at the maternal–foetal interface throughout gestation [13].

Maternal vitamin D physiology shifts upon conception. Circulating 1,25(OH)_2_D rises progressively across gestation, approximately doubling by the third trimester, a magnitude unmatched in any other physiological state. The increase reflects heightened renal CYP27B1 activity together with decidual and placental synthesis; decidual CYP27B1 expression is already prominent in the first trimester and, by several reports, higher in early than in late pregnancy [11], consistent with a role for locally generated 1,25(OH)_2_D in implantation and decidualisation rather than in late-gestation calcium handling. Vitamin D-binding protein (DBP), encoded by GC, rises in parallel under oestrogen control, with concentrations increasing by approximately 50–100% above pre-pregnancy values by the third trimester. Because approximately 85% of circulating 25(OH)D is bound to DBP, around 15% to albumin, and less than 1% circulates as free hormone, the dynamics of bioavailable 25(OH)D cannot be inferred from total 25(OH)D alone. The free hormone hypothesis holds that biologically active free fractions, rather than total 25(OH)D, drive tissue-level effects (Bikle, 1986) [13]. Boyd et al. (2026) recently applied this idea to offspring asthma, showing in a post hoc analysis of VDAART that estimated free 25(OH)D, but not DBP or total 25(OH)D individually, was significantly inversely associated with offspring asthma in mothers with asthma [9]. A temporal point deserves emphasis here: the active metabolite 1,25(OH)_2_D has a short circulating half-life of roughly 4–6 h, whereas the storage form 25(OH)D persists for approximately 2–3 weeks. Active hormone must therefore be generated continuously and locally, in the decidua, placenta, and kidney, rather than drawn from a stable circulating pool, which is precisely why decidual CYP27B1 capacity during the periconceptional weeks is mechanistically decisive.

The maternal–foetal interface is an immunologically privileged compartment in which semi-allogeneic foetal tissues must be tolerated by the maternal immune system. 1,25(OH)_2_D contributes to this tolerance through several converging mechanisms. Within the decidua, locally synthesised 1,25(OH)_2_D shifts the cytokine balance toward an anti-inflammatory profile, suppresses dendritic cell maturation, expands regulatory T cells, and modulates uterine natural killer (uNK) cell function [14]. Failure of decidual 1,25(OH)_2_D production has been implicated in recurrent miscarriage, with decreased VDR and CYP27B1 expression and lower decidual 25(OH)D concentrations documented in affected pregnancies [15]. Comparable defects have been described in pre-eclampsia, a disorder that shares with childhood asthma a hypothesised mechanistic root in altered maternal–foetal immune communication. The Hornsby et al. (2018) finding that maternal high-dose vitamin D supplementation enhances cord blood innate immune responsiveness, with upregulated TLR2 and TLR9 expression, places vitamin D within a coherent immunological framework that begins at the decidual interface and extends to the neonatal immune system at birth [16]. Aberrations in this framework during the periconceptional weeks, when the decidualised stroma is being established, and trophoblast invasion is in progress, plausibly carry long-reaching consequences.

Placental vitamin D handling also shapes foetal exposure. The foetus is endogenously incapable of 25-hydroxylation until the second half of gestation and is entirely dependent on placental transfer of maternal 25(OH)D, which crosses by passive diffusion in proportion to maternal concentrations, the bulk of foetal accrual occurring in the third trimester [17]. Cord blood 25(OH)D correlates strongly with maternal 25(OH)D at delivery, though the correlation with first-trimester maternal levels is weaker because of the long half-life of 25(OH)D (approximately 2–3 weeks) and intercurrent supplementation, season, and adiposity changes. VDR is expressed in cytotrophoblast, syncytiotrophoblast, and extravillous trophoblast across gestation, with target genes including those involved in invasion (matrix metalloproteinases), inflammation (NF-κB pathway elements), and trophoblast survival [13]. The Kawai et al. (2025) finding that low maternal second-trimester vitamin D is associated with epigenetic gestational age acceleration in offspring at birth [14] is plausibly mediated through placental epigenetic mechanisms, although the precise placental signatures remain to be characterised in human cohorts with serial sampling.

One implication is methodologically central. A single mid-pregnancy 25(OH)D measurement, the modal exposure variable in published cohort studies, captures neither the periconceptional environment in which implantation and early embryogenesis occurred, nor the dynamic free-hormone trajectory, nor the placental capacity for local 1,25(OH)_2_D synthesis. Exposure misclassification is therefore not a peripheral concern; it may be the central one.

## 3. The Periconceptional Window and Fetal Pulmonary Programming

**Figure 1:**
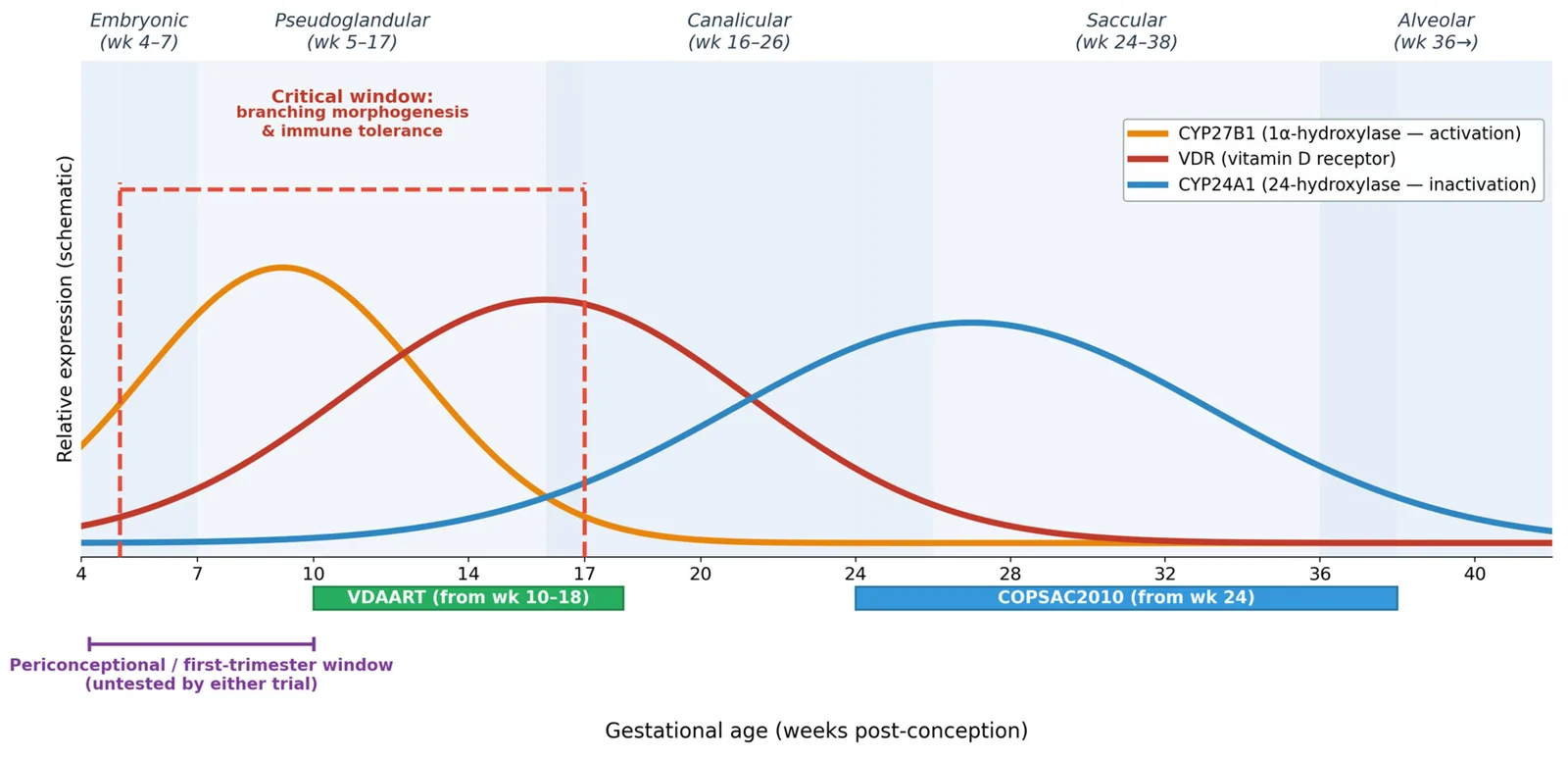
Developmental timeline of human pulmonary morphogenesis and schematic expression of vitamin D pathway genes. The five overlapping stages of lung development (embryonic, pseudoglandular, canalicular, saccular, alveolar) are shown against gestational age. Schematic relative-expression curves indicate the early decidual and pulmonary prominence of CYP27B1 (1α-hydroxylase, activation), the broad and sustained expression of VDR, and the later rise in CYP24A1 (24-hydroxylase, inactivation). The dashed box marks the critical window of branching morphogenesis and immune-tolerance establishment (approximately weeks 5–17). Horizontal bars show the intervention windows of the two pivotal trials, VDAART (from weeks 10–18) and COPSAC2010 (from week 24), both beginning after the periconceptional and early first-trimester interval (bracketed), which neither trial targeted. Expression profiles are schematic, drawn from developmental transcriptomic evidence, and are intended to convey temporal relationships rather than absolute quantities.

Human pulmonary morphogenesis unfolds in five overlapping stages. The embryonic phase spans weeks 4–7 post-conception; the pseudoglandular phase extends from week 5 to week 17 and establishes the conducting airway tree down to the terminal bronchiole; the canalicular phase occupies weeks 16–26; the saccular phase covers weeks 24–38; the alveolar phase begins around week 36 and continues postnatally into early childhood [9,18]. Branching morphogenesis, the architectural template of the conducting airways, is therefore largely complete before any extant prenatal vitamin D trial has enrolled its first participant. The temporal relationship between these stages, the expression of the principal vitamin D pathway genes, and the intervention windows of the two pivotal trials is summarised in Figure 1.

Kho et al. (2013) screened the transcriptomes of developing mouse and human foetal lungs and identified a network of vitamin D pathway genes that are temporally regulated during pulmonary morphogenesis and that overlap substantially with known asthma susceptibility loci [15]. Their analysis included VDR, CYP24A1 (encoding the inactivating 24-hydroxylase), CYP27B1, and several downstream effectors, indicating a developmentally tight window during which vitamin D signalling integrates with airway patterning [19]. The inference from such data nonetheless requires caution. Much of the developmental transcriptomic evidence derives from murine models, and the timing of pulmonary morphogenesis differs between species: mice are born in the saccular stage, whereas human neonates have entered early alveolarisation. Direct human protein-level confirmation of VDR expression in the first-trimester lung is sparse. Transcriptomic signals would be strengthened by immunohistochemical or tissue-profiling data, for example, from the GTEx or Human Protein Atlas resources, establishing where and when the receptor protein is present during early human airway development.

Beyond direct transcriptional regulation, vitamin D acts as an epigenetic modifier in early development. Anderson et al. (2018), in a randomised pilot of 3800 IU/day versus 400 IU/day cholecalciferol from late second trimester through 4–6 weeks postpartum, reported that maternal supplementation altered DNA methylation in 217 cytosine-guanine sites in breastfed infants at 4–6 weeks, with differential methylation mapping most strongly to collagen metabolic processes and regulation of apoptosis [20]. Kawai et al. (2025), in a Japanese cohort, reported that low maternal serum vitamin D in the second trimester was negatively associated with epigenetic gestational age acceleration in offspring at birth (regression coefficient −0.022, 95% CI −0.039 to −0.005, by Bohlin’s method), a marker of developmental programming independent of chronological gestational age [14]. A central interpretive caveat applies to both studies: neither demonstrates that the differentially methylated sites are functional, that is, whether they alter gene expression in a direction relevant to the respiratory phenotype. Population-level methylation studies are further constrained by cell-type heterogeneity in the sampled tissue and by the absence of paired transcriptomic data, so the mechanistic link between maternal 25(OH)D and the infant methylome remains imperfectly mapped, particularly for the periconceptional weeks; existing studies have largely sampled the second trimester onward.

A separate strand of evidence implicates the maternal metabolome. Kim et al. (2023), in a combined COPSAC2010–VDAART metabolomics analysis, identified the sphingomyelin pathway as significantly enriched among metabolites distinguishing mothers with higher gestational 25(OH)D, and showed that a 46-metabolite vitamin D-related profile at 1 week postpartum was associated with reduced offspring recurrent wheeze (HR 0.92, 95% CI 0.86–0.98) and wheeze exacerbations (HR 0.90, 95% CI 0.84–0.97) in early childhood [21]. The sphingomyelin signal positions vitamin D within a biologically coherent metabolic milieu rather than as a stand-alone hormonal exposure, and aligns with the role of *ORMDL3* (a negative regulator of serine palmitoyltransferase at the 17q21 asthma locus) in sphingolipid synthesis. A mechanistic chain can be proposed in which adequate vitamin D status modulates the maternal sphingolipid pool, attenuating the *ORMDL3*-driven dysregulation of sphingolipid metabolism that has been linked to bronchial hyperresponsiveness; however, this sequence remains speculative and awaits direct mechanistic testing rather than inference from correlated metabolomic and genetic signals.

Human observational data bear directly on the consequences of first-trimester deficiency. Lee et al. (2025), in a Korean retrospective cohort of 5169 women with serial first- and second-trimester 25(OH)D measurements, found that first-trimester deficiency was associated with elevated risks of preterm birth before 34 weeks (aOR 2.42, 95% CI 1.24–4.71) and developmental delay (aOR 4.46, 95% CI 2.41–8.27); these risks were not attenuated when second-trimester 25(OH)D was normalised through supplementation [19]. The pattern, in which first-trimester deficiency imprints outcomes despite later correction, is the empirical fingerprint of a critical window that closes early. Whether the same logic applies to airway programming has not been directly tested in cohorts with periconceptional sampling. Indeed, direct data on vitamin D status during gestational weeks 0–10, the interval of primary bronchial branching, are essentially absent from the literature; this gap is not a marginal omission but the central unaddressed question, and we position it here as the priority direction for future cohort design rather than as a settled premise.

Hornsby et al. (2018) brought immunological evidence into the field, showing in a VDAART cord blood sub-study that maternal 4400 IU/day cholecalciferol enhanced the proinflammatory response of cord blood mononuclear cells to innate and mitogenic stimuli, with upregulated *TLR2* and *TLR9* expression, consistent with priming of neonatal innate immunity at the time of birth [16]. Vitamin D exposure during foetal development thus appears to shape both the structural template of the airway and the immunological responsiveness it will encounter postnatally.

## 4. Observational Evidence Across Pregnancy Trimesters

The observational literature on maternal vitamin D and childhood asthma is voluminous and divergent. Vitamin D deficiency, defined as serum 25(OH)D below 50 nmol/L (20 ng/mL), is highly prevalent among pregnant women worldwide [22,23]. Bodnar et al. (2007) documented deficiency or insufficiency at delivery in 83% of Black and 47% of White women in a northern US cohort, with corresponding deficiency in their neonates [24]. Regional European data are consistent: a Romanian prospective cohort of women delivering at 32 weeks or earlier reported vitamin D deficiency or severe deficiency in roughly three-quarters of mothers, a strong maternal–neonatal 25(OH)D correlation (r = 0.68), and an independent association of cold-season birth with severe maternal deficiency [25]. Beyond respiratory outcomes, maternal deficiency has been associated in meta-analysis with increased risks of pre-eclampsia, gestational diabetes, and small-for-gestational-age birth [26,27]. The magnitude of these associations is clinically appreciable, with the meta-analytic evidence indicating a significantly higher risk of pre-eclampsia and small-for-gestational-age birth in deficient than in replete women, and a weaker, less consistent association with gestational diabetes, though the causal strength varies across outcomes and populations, and residual confounding is difficult to exclude [27]. For childhood respiratory outcomes specifically, Pacheco-González et al. (2018), aggregating 34 observational studies, found an inverse association between higher prenatal 25(OH)D and offspring respiratory tract infections, and a positive association with school-age FEV_1_ z-score; the association with asthma, wheeze, eczema, and allergic sensitisation did not reach conventional significance [28]. Methodological heterogeneity dominates the field [22,29]: cohorts differ in 25(OH)D assay (radioimmunoassay versus liquid chromatography–tandem mass spectrometry, the latter now considered the gold standard), in the trimester of sampling, in latitude, in the season distribution of pregnancies, in the definition and ascertainment of asthma, and in the age of outcome assessment.

A point that recurs across these analyses, and that is rarely emphasised in syntheses, is that nearly all sampling occurred from the second trimester onward. Studies with first-trimester 25(OH)D measurement remain rare, and studies with preconceptional measurement are nearly non-existent outside small fertility cohorts. The exposure window most consistently sampled in observational research is therefore not the window most plausibly relevant for airway programming.

A second methodological caveat concerns DBP and free 25(OH)D. Most cohorts measured total 25(OH)D without simultaneous DBP. Boyd et al. (2026), in a post hoc analysis of 518 VDAART mother–child pairs, demonstrated that DBP modified the effect of total 25(OH)D on offspring asthma risk: in mothers with asthma, estimated free 25(OH)D was significantly inversely associated with offspring asthma at 3 years, outperforming both total 25(OH)D and DBP alone [9]. The implication is that observational studies measuring only total 25(OH)D have underestimated the relevant exposure in a non-random fashion correlated with maternal genotype and asthma status.

A third caveat concerns confounding. Maternal vitamin D status correlates with maternal socioeconomic status, diet, body mass index, smoking, ethnicity, latitude, season, and outdoor physical activity, each of which independently predicts childhood respiratory outcomes. Even with adjustment, residual confounding in observational data is substantial. The honest conclusion is that observational evidence supports a plausible inverse signal but cannot establish causality.

## 5. Trial Evidence and the Limits of Late-Window Intervention

The two pivotal randomised trials, VDAART and COPSAC2010, were designed in parallel and published their primary outcomes simultaneously in 2016; their designs and principal findings are summarised in Table 1. Litonjua et al. randomised 881 US pregnant women at high asthma-related risk (own or partner history) to 4400 IU/day cholecalciferol (4000 IU plus a 400 IU standard prenatal multivitamin) versus 400 IU/day, from weeks 10–18 to delivery; with 806 mother–child pairs analysed, the primary outcome of asthma or recurrent wheeze by 3 years showed a hazard ratio of 0.8 (95% CI 0.6–1.0, *p* = 0.051) [30]. Chawes et al. randomised 623 Danish women, from a population-based birth cohort, to 2400 IU/day cholecalciferol or a matching placebo on top of the standard 400 IU/day, from week 24 of gestation to one week postpartum, for a total of 2800 IU/day in the active arm. Persistent wheeze was diagnosed in 47/315 children (16%) in the active arm and 57/308 (20%) in the placebo arm (hazard ratio 0.76, 95% CI 0.52–1.12, *p* = 0.16) [4].

The combined patient-level analysis by Wolsk et al. (2017) crystallised the signal [5]. The pooled adjusted odds ratio for offspring asthma or recurrent wheeze at 0–3 years was 0.74 (95% CI 0.57–0.96, *p* = 0.02). Stratification by baseline 25(OH)D was revealing: in women with entry 25(OH)D ≥ 30 ng/mL, the odds ratio fell to 0.54 (95% CI 0.33–0.88, *p* = 0.01); in women with entry levels < 30 ng/mL, no significant effect emerged (aOR 0.84, 95% CI 0.62–1.15) [31]. The interpretation is biologically uncomfortable but consistent: supplementation worked best in women who were already replete at randomisation. A plausible reading is that women starting from replete status had been replete for longer, plausibly through conception, and that the benefit reflects a longer-duration exposure rather than the trial supplementation itself. It is worth stating the direction of this effect modification plainly, since it is easily misread: the clear benefit appeared in women replete at entry, not in those who were deficient. Rather than implying that supplementation is superfluous in deficiency, this pattern points to sustained repletion across conception, which a replete-at-entry status approximates, as the relevant exposure. The corollary is that deficient women may require repletion to begin before conception for any comparable benefit to emerge.

The 6-year follow-ups dampened enthusiasm. Litonjua et al. (2020) reported no statistically significant difference between treatment arms in cumulative incidence of asthma or recurrent wheeze through age 6 in the intention-to-treat analysis [30]. Brustad et al. (2019) reported a similar absence of effect at 6 years in COPSAC2010 [32]. The standard interpretation has been that the early effect represents prevention of transient preschool wheeze, largely viral-induced and amenable to immunomodulation, rather than of stable, atopy-associated school-age asthma. An alternative interpretation, less frequently advanced, is that mid-gestational supplementation acts on a developmental substrate that has already been patterned, producing a transient functional benefit without altering the underlying disease trajectory.

The 15-year analysis from VDAART revisited the data with two strategies. First, an intention-to-treat analysis adjusted for entry 25(OH)D, in which the supplementation effect on asthma at 3 and 6 years recovered statistical significance. Second, stratification by maternal genotype at 17q21 rs12936231, a functional single-nucleotide polymorphism that modulates *ORMDL3* expression and is the most robustly replicated childhood asthma susceptibility locus, revealed that the protective effect of supplementation was confined to GG/GC genotype combinations [8]. Mirzakhani et al. (2021) had earlier extended this observation: in both VDAART and COPSAC2010, offspring of mothers with the low-risk GG or GC genotype receiving high-dose vitamin D had a significantly reduced risk of asthma/recurrent wheeze (HR 0.54, 95% CI 0.37–0.77, *p* < 0.001 for VDAART; HR 0.56, 95% CI 0.35–0.92, *p* = 0.021 for COPSAC2010), whereas no effect was observed in offspring of mothers with the high-risk CC genotype (HR 1.05, 95% CI 0.61–1.84 for VDAART; HR 1.11, 95% CI 0.54–2.28 for COPSAC2010) [33]. Shadid et al. (2023), also in the VDAART cohort, showed that the protective effect was maximal when supplementation began earliest within the allowed enrolment window (closer to week 10 than week 18), supporting a temporal interpretation [34].

The 2025 Cochrane review by Patchen et al., aggregating randomised trials of vitamin D in pregnant or lactating women or in early childhood, concluded that high-dose vitamin D in pregnancy likely reduces childhood wheeze (RR 0.79, 95% CI 0.64–0.98; three studies, 1439 participants; moderate-certainty evidence) but likely produces little to no difference in childhood asthma per se (RR 0.81, 95% CI 0.63–1.04; two studies, 1355 participants; moderate-certainty evidence) [6]. The 2025 meta-analysis by Li et al. (seven RCTs, 3958 mother–infant pairs) found no significant overall effect on respiratory tract infections, asthma, wheezing, eczema, or allergic outcomes [7]. Shen et al. (2025) reached convergent conclusions [8]. The systematic review by Svensson et al. (2025), focusing on offspring health outcomes from 10 days postpartum and beyond, concluded that 11 of 16 studies showed reduced respiratory tract infections in the first years of life, but that even 1600 IU/day during pregnancy was associated with a high frequency of infant vitamin D insufficiency at birth [22].

A coherent reading of these results requires distinguishing between two negative findings. The first is the failure of a moderate dose, started in mid-pregnancy, to durably alter offspring asthma incidence in unselected populations, a conclusion now well established. The second is the broader question of whether vitamin D in early human development has any causal role in asthma at all. The latter question is not resolved by the former: the trials that have been conducted constitute weak evidence against the periconceptional hypothesis because they were not designed to test it.

**Table 1:** Randomised trials of prenatal vitamin D supplementation and childhood respiratory outcomes.

Trial (First Author, Year)	Population and Dose	Onset of Intervention	Primary Outcome (ITT)	Long-Term Findings
VDAART (Litonjua, 2016) [3]	n = 881 US, high-risk (analysed n = 806); 4400 vs. 400 IU/d	Weeks 10–18	Asthma/wheeze at 3 y: HR 0.8 (0.6–1.0), *p* = 0.051	No effect at 6 y ITT (Litonjua, 2020) [30]; recovered with baseline-adjustment and 17q21 stratification at 15 y
COPSAC2010 (Chawes, 2016) [4]	n = 623 Danish, unselected; 2800 IU/d total (2400 added to standard 400) vs. placebo + 400 IU/d	Week 24	Persistent wheeze at 3 y: HR 0.76 (0.52–1.12), *p* = 0.16	No effect at 6 y ITT (Brustad, 2019) [32]; modified by VDR genotype
Goldring (2013) [35]	n = 180 UK, ethnically stratified; 800 IU/d ergocalciferol (D_2_) daily or single 200,000 IU cholecalciferol (D_3_) bolus vs. no supplement	Week 27	No effect on wheeze at 3 y (RR 0.86, 95% CI 0.49–1.50)	–
MAVIDOS (Cooper, 2016) [36]	n = 1134 UK; 1000 IU/d cholecalciferol vs. placebo (bone outcomes; respiratory outcomes not assessed)	Week 14	Neonatal BMC: no overall effect; benefit in prespecified winter-born subgroup	Greater offspring areal bone mineral density at age 4 y in single-centre follow-up (Curtis 2022) [37]

Abbreviations: BMC, bone mineral content; CI, confidence interval; HR, hazard ratio; ITT, intention-to-treat; RR, risk ratio; VDR, vitamin D receptor.

## 6. Effect Modifiers: Towards a Stratified Approach

The repeated finding that average treatment effects are small or null while subgroup effects are large is the empirical signature of effect modification. For prenatal vitamin D, several modifiers have been identified, each with a distinct biological rationale.

Baseline 25(OH)D status emerges as the most consistent modifier. Across both VDAART and COPSAC2010, women entering with 25(OH)D ≥ 30 ng/mL derived substantially greater benefit than those entering with deficient status [8,31]. The interpretation is non-obvious. One reading frames the effect as a threshold phenomenon: only women who maintain replete status throughout the periconceptional and first-trimester period, rather than only during the trial, deliver foetuses to a permissive epigenetic environment. A competing reading frames it as a duration effect: women already replete at week 10 were plausibly replete before conception, so the effective exposure window is longer in this group.

Maternal genotype at 17q21 rs12936231 modifies the effect of supplementation across both VDAART and COPSAC2010, as shown by Mirzakhani et al. (2021) [33]. The 17q21 locus contains *IKZF3*, *ZPBP2*, *ORMDL3*, and *GSDMB*; rs12936231 is a regulatory variant influencing *ORMDL3* expression. The C allele is associated with higher *ORMDL3* expression and with increased asthma risk, particularly for early-onset, virus-induced asthma. The mechanism by which vitamin D supplementation appears protective specifically in non-CC carriers remains incompletely characterised but may involve vitamin D modulation of sphingolipid synthesis, the same axis identified in maternal metabolomics by Kim et al. [21], and the role of the ORMDL3 protein as a regulator of serine palmitoyltransferase.

Variants in vitamin D pathway genes have also been investigated. Brustad et al. (2021) showed that the effect of high-dose supplementation in COPSAC2010 was modified by VDR genotype at rs1544410, with the largest effect in offspring of mothers with the TT genotype (HR 0.26, 95% CI 0.10–0.68, *p* = 0.006) and no effect in CT or CC genotypes [38]. The VDR interaction did not replicate in VDAART, and no significant effect modification from maternal or offspring GC genotype was observed in either cohort [39]. The Boyd et al. (2026) analysis demonstrated that DBP modified the effect of total 25(OH)D on offspring asthma risk, with significant joint effects in mothers without asthma and a significant inverse association between estimated free 25(OH)D and offspring asthma in mothers with asthma [9].

Maternal asthma status emerges as an effect modifier in its own right. In the Boyd et al. analysis, the inverse association between estimated free 25(OH)D and offspring asthma was confined to mothers with asthma [9]. The result aligns with the broader observation that maternal asthma alters maternal–foetal immune communication and that high-risk pregnancies may benefit disproportionately from interventions targeting that communication.

Ethnicity and skin pigmentation modify cutaneous vitamin D synthesis and remain understudied modifiers of the supplementation effect. Both VDAART and COPSAC2010 were predominantly conducted in light-skinned populations of European ancestry, despite the substantially higher prevalence of vitamin D deficiency in pregnant women of African, South Asian, and Hispanic backgrounds. The disparity in prevalence may translate into disparity in attributable benefit, but this has not been adequately quantified.

Season of conception is a final, often overlooked modifier. Latitude determines the months during which cutaneous 25(OH)D synthesis is possible; at latitudes above approximately 35°, UVB-driven synthesis is minimal from October to April. A woman conceiving in late autumn at 47 °N is likely to enter the first trimester deficient unless supplemented, regardless of mid-pregnancy supplementation policy. The interaction between season of conception, latitude, and supplementation timing was recognised but rarely modelled explicitly.

The prenatal exposome shapes maternal vitamin D availability in ways that interact with supplementation efficacy. Maternal adiposity sequesters lipophilic 25(OH)D in adipose tissue, lowering bioavailable concentrations at a given oral dose. Wortsman et al. (2000) showed that the incremental rise in serum vitamin D_3_ after whole-body UV irradiation is 57% lower in obese than in lean individuals [26]. Cohort and trial data consistently report attenuated 25(OH)D responses to supplementation in women with higher pre-pregnancy body mass index (BMI), while the offspring of obese women exhibit elevated asthma risk independent of vitamin D status, suggesting parallel pathways that may converge mechanistically. Maternal smoking is associated with lower circulating 25(OH)D: a meta-analysis of 24 studies and 11,340 participants found consistently lower concentrations in smokers than non-smokers, with proposed mechanisms including cadmium accumulation and downregulation of CYP27B1 in airway epithelium [37]. Endocrine-disrupting chemicals, particularly phthalates and bisphenols, have been shown to disrupt circulating 25(OH)D concentrations during pregnancy and to modulate VDR signalling, with documented downstream associations to preterm birth and pre-eclampsia [40]. Ambient air pollution has been associated independently with lower maternal 25(OH)D; a meta-analysis of gestational exposure reported higher odds of suboptimal vitamin D per increment of PM_2_._5_ (odds ratio 1.43 per 5 µg/m^3^, 95% CI 1.02–1.99), an effect mediated substantially by reduced cutaneous UV-B exposure [41]. Gestational weight gain, dietary patterns, and seasonality further modulate the trajectory of maternal 25(OH)D across pregnancy. The implication is that vitamin D status cannot be analysed in isolation; the prenatal exposome conditions both the exposure variable and the outcome, and trials that adjust only for measured 25(OH)D will systematically underestimate the modifier-conditional effect.

Taken together, these modifiers articulate an emerging precision–prevention framework: the women in whom periconceptional vitamin D optimisation is most likely to alter offspring outcomes are those with deficient baseline status, a permissive maternal–foetal genotype combination, maternal asthma history, and conception in autumn or winter at temperate latitudes, particularly when superimposed exposome burdens (obesity, smoking, urban pollution) further compromise the vitamin D axis. The unselected average effect, which most published meta-analyses now estimate as modest or null, conceals this stratified picture.

## 7. Translational Implications and Current Recommendations

Current guidelines reflect the unsettled state of the evidence. The 2024 Endocrine Society clinical practice guideline by Demay et al. recommends empiric vitamin D supplementation during pregnancy without routine 25(OH)D testing, citing potential reductions in pre-eclampsia, intra-uterine mortality, preterm birth, small-for-gestational-age birth, and neonatal mortality [42]. It does not specify a dose and does not address the preconceptional interval. It explicitly recommends against routine 25(OH)D measurement, a position subsequently challenged on the grounds that a one-size-fits-all approach without monitoring cannot reliably achieve the threshold (≥30 ng/mL [≥75 nmol/L], on the available evidence) at which most subgroup effects emerge, and that precision-guided screening is warranted in deficiency-prone groups, including pregnant women [39].

No major international body currently recommends preconceptional 25(OH)D screening or routine supplementation in women planning pregnancy, despite the precedent set by folic acid for neural tube defect prevention. Folate supplementation was introduced into preconceptional care because the developmental window for neural tube closure (days 21–28 post-conception) precedes pregnancy recognition; vitamin D supplementation, if it acts at all on offspring respiratory programming, faces an analogous timing constraint. Framing the question within the first 1000 days reinforces this logic: interventions delivered in the earliest segment of that window, before and around conception, target developmental processes that later supplementation cannot reach [24,43,44].

The major guidance documents diverge on dose and on whether status should be measured, and none addresses the preconceptional interval directly (Table 2). This divergence reflects genuine evidential uncertainty rather than disagreement over the underlying biology.

Practical implications for clinical care, advanced cautiously and recognising the unsettled evidence, would include the following. First, women planning pregnancy and presenting for preconceptional counselling could reasonably be screened for 25(OH)D status, particularly if they belong to deficiency-prone groups: dark skin, veiled clothing, obesity, malabsorption, or autumn or winter conception planned at temperate latitudes. Second, women found to be deficient could be supplemented to a target of ≥30 ng/mL, the threshold above which the post hoc benefit signal in the trials emerged, using doses of 1000–2000 IU/day cholecalciferol, with rescreening at 8–12 weeks. Third, supplementation should be continued throughout pregnancy and lactation rather than being discontinued at delivery, given the ongoing relevance of postnatal vitamin D for both maternal and infant health. Fourth, none of these recommendations should be presented to patients as established preventive care for offspring asthma; the appropriate framing is bone, calcium, and broader maternal–neonatal benefit, with the asthma signal acknowledged as biologically plausible but not yet definitively established.

On dose, MAVIDOS (Cooper et al., 2016) demonstrated that 1000 IU/day cholecalciferol from week 14 was sufficient to achieve repletion in most participants and was safe; although its primary neonatal bone outcome was null across the whole cohort, a prespecified single-centre follow-up reported greater offspring areal bone mineral density at age 4 years [36]. VDAART used 4400 IU/day total, and COPSAC2010 used 2800 IU/day total, both without safety signals. The systematic review by Svensson et al. (2025) noted that even 1600 IU/day during pregnancy was associated with a high frequency of infant vitamin D insufficiency at birth in some series [22]. The supplementation envelope is therefore broad, and the appropriate dose for preconceptional or first-trimester use is more likely to be governed by titration to target 25(OH)D than by a fixed value.

A question raised repeatedly in appraisals of this literature is whether supplementation is better initiated before or during pregnancy, and how baseline status conditions any benefit. The trial evidence is indirect but suggestive. Neither VDAART nor COPSAC2010 enrolled on the basis of vitamin D deficiency, and neither targeted the preconceptional interval: supplementation began at weeks 10–18 and week 24, respectively, after the embryonic and much of the pseudoglandular phase of airway morphogenesis. The Wolsk et al. (2017) finding that benefit concentrated in women already replete at randomisation (entry 25(OH)D ≥30 ng/mL) is therefore most coherently read as a proxy signal: women replete at week 10 had plausibly been replete through conception, so the apparent benefit reflects a longer effective exposure that already encompassed the periconceptional weeks [5]. Lee et al. (2025) reinforce this reading from the observational side, showing that first-trimester deficiency imprinted adverse perinatal and developmental outcomes that normalising second-trimester 25(OH)D did not reverse [19]. On the available evidence, the distinction is not pre-conception versus in-pregnancy as mutually exclusive options, but rather whether repletion is achieved and sustained across the periconceptional window; supplementation that begins only in mid-pregnancy cannot, by construction, secure that window.

Other micronutrients, including vitamin E, beta-carotene, and the long-chain omega-3 fatty acids, have likewise been linked to offspring’s allergic and respiratory outcomes, which raises the question of why vitamin D should be privileged. The answer lies in mechanism and timing rather than in any claim of unique importance. Vitamin E and carotenoids act largely as antioxidants modulating the postnatal oxidative and immune milieu, and the omega-3 fatty acids exert their best-characterised effects on the lipid-mediator landscape during late pregnancy, lactation, and early complementary feeding. Vitamin D, by contrast, operates through receptor-mediated transcriptional control, local decidual 1,25(OH)_2_D synthesis, and epigenetic programming that are concentrated in the periconceptional and first-trimester interval. These nutrients are therefore complementary rather than interchangeable, and pooling them in a single analysis would conflate distinct pathways acting in distinct windows. A corollary follows directly: vitamin D supplementation cannot compensate for a broadly inadequate maternal diet. Where deficiencies of iron, folate, protein, or the antioxidant vitamins coexist, correcting 25(OH)D alone is unlikely to normalise offspring risk, and the periconceptional vitamin D strategy set out above should be understood as one element of comprehensive preconceptional nutritional optimisation, not as a substitute for it.

Several factors constrain supplementation as a population strategy, and they bear on the wider point that supplementation cannot simply offset an established deficiency. Adiposity sequesters lipophilic 25(OH)D, so that a fixed oral dose produces a smaller increment in women with higher BMI; Wortsman et al. (2000) quantified a 57% lower incremental rise after standardised UV exposure in obese compared with lean individuals [26]. Genetic variation at GC, VDR, and the 17q21 locus modifies both the bioavailable fraction of a given dose and the downstream response to it [1,39,45], so that uniform dosing yields non-uniform tissue exposure. Season and latitude impose a further constraint: at temperate latitudes, cutaneous synthesis is negligible for much of the year, so a woman conceiving in autumn may enter the first trimester deficient regardless of mid-pregnancy policy. Titration to a target 25(OH)D, rather than a fixed dose, is therefore more defensible on current evidence, and the appropriate dose for preconceptional or first-trimester use is more likely to be governed by achieved concentration than by a single prescribed value.

## 8. Limitations and a Research Agenda

Several limitations of the current evidence base deserve explicit articulation, both to discipline the interpretation of existing data and to delineate the research that remains necessary.

25(OH)D assays differ. Radioimmunoassay, chemiluminescent immunoassay, and liquid chromatography–tandem mass spectrometry each yield slightly different absolute values, with material inter-method variability across cohort eras. Therefore, cross-study aggregation in meta-analyses introduces unquantified noise that may obscure modest effects.

Asthma is not a single disease. Preschool wheeze and school-age asthma may share clinical features but represent partially distinct endotypes, with different genetic and environmental determinants. Most trials and cohorts have aggregated outcomes without distinguishing between transient early wheeze, persistent wheeze, atopic asthma, and viral-triggered asthma. The COPSAC2010 phenotyping approach, with detailed clinical assessment by trained paediatricians and objective measures, is the methodological standard but is rarely replicated.

Sample timing, as discussed throughout this review, is biassed toward the second and third trimesters. Cohorts with serial sampling from preconception through delivery are required to determine whether the periconceptional window is independently informative beyond mid-pregnancy 25(OH)D. Such cohorts exist for fertility outcomes, but rarely link to childhood respiratory follow-up.

DBP and free 25(OH)D have not been routinely measured. The Boyd et al. (2026) analysis demonstrated that omitting these measurements introduces material misclassification, particularly across *GC* haplotypes [9]. Future studies should incorporate direct measurement of DBP and, where feasible, of free 25(OH)D, alongside total 25(OH)D.

Geographic and ancestral representation remains narrow. Mediterranean and Eastern European populations, including those at latitudes of high winter prevalence of deficiency, are under-represented in the existing trial evidence. Regional cohorts, including in Romania, where the combination of latitude (44–48° N), continental climate, and an underdocumented baseline 25(OH)D status creates conditions of particular interest, could materially extend the generalisability of findings.

A research agenda follows from these limitations. The single highest-value investment is a preconceptional randomised controlled trial: randomisation with at least 12 weeks of supplementation before attempted conception, to high-dose cholecalciferol or placebo, with serial 25(OH)D and DBP measurement, comprehensive maternal and offspring genotyping at vitamin D pathway and asthma susceptibility loci, cord blood metabolomics and methylomics, and follow-up to at least 6 and ideally 10 years with objective phenotyping including spirometry, oscillometry and fractional exhaled nitric oxide. Such a trial would also address the methodological criticism that mid-pregnancy enrolment cannot test the periconceptional hypothesis. Complementary work should include refinement of the metabolomic signatures that mediate vitamin D effects on offspring asthma and mechanistic studies in human pluripotent stem cell-derived lung organoids to dissect the developmental windows of vitamin D sensitivity that are inaccessible to clinical investigation.

## 9. Concluding Remarks

The evidence accumulated since 2016 supports a coherent, if uncomfortable, picture. Maternal vitamin D supplementation initiated in mid-pregnancy produces a modest, often statistically marginal, short-term reduction in offspring asthma or recurrent wheeze that does not persist to school age in unselected populations. Post hoc analyses recover meaningful effects when stratified by baseline 25(OH)D and maternal genotype, suggesting that a real biological signal exists but is masked at the population level. Mechanistic and developmental biology converge on the periconceptional and first-trimester window as the period during which the offspring’s respiratory phenotype is most plausibly programmable.

The most consequential implication is methodological rather than therapeutic. Future trials designed to test the periconceptional hypothesis, rather than to extend an already exhausted mid-pregnancy paradigm, are needed. In parallel, guidelines could reasonably move toward routine preconceptional 25(OH)D screening and targeted repletion in deficient women, framed within the broader maternal–neonatal benefit envelope and without overstating the offspring asthma evidence. The folate precedent shows that preconceptional nutritional interventions can be implemented at a population scale once the developmental rationale is established; vitamin D is not yet at that point for asthma prevention, but the biological case for moving the question upstream has become difficult to set aside.

## Figures and Tables

**Table 2:** Comparison of recommendations on vitamin D in pregnancy across major guideline bodies.

Body (Year)	Routine 25(OH)D Screening	Supplementation in Pregnancy	Notes
WHO (2020)	Not recommended	Not recommended as routine antenatal care; where deficiency is documented, 200 IU/day (aligned to dietary intake)	Conditional recommendation; vitamin D plus calcium showed little or no effect on most outcomes
Endocrine Society (2024)	Recommended against (routine testing or follow-up)	Empiric supplementation suggested; trials ranged 600–5000 IU/day, weighted average ≈2500 IU/day	Cites reductions in pre-eclampsia, preterm birth, SGA, and intra-uterine/neonatal mortality; no dose specified
ACOG (2011, reaffirmed)	Not recommended universally; consider in high-risk women	Prenatal vitamin (≈400–600 IU/day) for low risk; 1000–2000 IU/day where deficiency is identified	Up to 4000 IU/day is regarded as safe; awaits completion of RCTs
IOM/NAM (2011)	Not addressed	Adequate intake 600 IU/day; tolerable upper limit 4000 IU/day	Dietary reference intake based on skeletal endpoints, not pregnancy-specific outcomes

Abbreviations: ACOG, American College of Obstetricians and Gynecologists; IOM/NAM, Institute of Medicine/National Academy of Medicine; IU, international units; SGA, small for gestational age; WHO, World Health Organization. None of the listed bodies issues a recommendation specific to the preconceptional period.

## Data Availability

No new data were created or analyzed in this study. Data sharing is not applicable to this article.
